# Pre-fertilization gamete thermal environment influences reproductive success, unmasking opposing sex-specific responses in Atlantic salmon (*Salmo salar*)

**DOI:** 10.1098/rsos.231427

**Published:** 2023-12-13

**Authors:** Marco Graziano, Monica F. Solberg, Kevin A. Glover, Ramakrishnan Vasudeva, Lise Dyrhovden, David Murray, Simone Immler, Matthew J. G. Gage

**Affiliations:** ^1^ Centre for Ecology, Evolution, and Conservation, School of Biological Sciences, University of East Anglia, Norwich NR4 7TJ, UK; ^2^ Population Genetics Group, Institute of Marine Research, 5817 Bergen, Norway; ^3^ Centre for Environment Fisheries and Aquaculture Science (CEFAS), Lowestoft NR33 0HT, UK; ^4^ Faculty of Biological Sciences, School of Biology, University of Leeds, Leeds LS2 9JT, UK

**Keywords:** climate change, thermal plasticity, reproductive function, sperm damage, sex-specific responses, external fertilizers

## Abstract

The environment gametes perform in just before fertilization is increasingly recognized to affect offspring fitness, yet the contributions of male and female gametes and their adaptive significance remain largely unexplored. Here, we investigated gametic thermal plasticity and its effects on hatching success and embryo performance in Atlantic salmon (*Salmo salar*). Eggs and sperm were incubated overnight at 2°C or 8°C, temperatures within the optimal thermal range of this species. Crosses between warm- and cold-incubated gametes were compared using a full-factorial design, with half of each clutch reared in cold temperatures and the other in warm temperatures. This allowed disentangling single-sex interaction effects when pre-fertilization temperature of gametes mismatched embryonic conditions. Pre-fertilization temperature influenced hatch timing and synchrony, and matching sperm and embryo temperatures resulted in earlier hatching. Warm incubation benefited eggs but harmed sperm, reducing the hatching success and, overall, gametic thermal plasticity did not enhance offspring fitness, indicating vulnerability to thermal changes. We highlight the sensitivity of male gametes to higher temperatures, and that gamete acclimation may not effectively buffer against deleterious effects of thermal fluctuations. From an applied angle, we propose the differential storage of male and female gametes as a tool to enhance sustainability within the hatcheries.

## 1. Introduction

Environmental changes are intrinsically related to key evolutionary concepts including adaptation, selection and speciation. Irrespective of the causes and strengths of environmental changes, they inevitably influence natural populations; the efficiency and degree of resistance of the latter depend on their ability to adjust through adaptive responses such as migration, phenotypic plasticity, and epigenetics [1,2]. These mechanisms determine the long-term perspective of organisms persisting in the face of climate change [3]. The ability to evolve an adaptive response strongly depends on genetically heritable variation and on the demographic costs of such adaptation [4]. If environmental changes are too extreme or if they occur over periods that are evolutionarily irrelevant, high mortality and extinction will prevent any adaptive evolutionary response [5]. Here, phenotypic plasticity may aid in the expression of differential phenotypes and allow for an efficient strategy to cope with sudden environmental fluctuations [6].

Phenotypic plasticity in the context of environmental change is particularly important when it affects several generations, referred to as inter- or transgenerational plasticity, where the environment or condition of the parents affects the fitness of future generation(s) [7–9]. Such multi-generational responses can be adaptive when the environment experienced by the parent/s is a good predictor for the conditions to be encountered by the offspring, enhancing its fitness in the matched environment [10–12]. In the seed beetle *Stator limbatus*, mothers raised under poor nutritional conditions pass on their environmental ‘experience’ by increasing egg size to protect their offspring against starvation [13]. In the flour beetle *Tribolium castaneum*, males that experienced an experimental heat wave produced sons whose reproductive fitness remained lower than controls despite experiencing benign conditions, demonstrating a sire effect carried over to the offspring via the sire's sperm [14]. In a follow up study using the same model organism, a single generation exposure at 30°C or 38°C revealed that both sperm and ova were optimized (in opposing directions) for their morphology and function in the environment in which the plasticity was induced, revealing an adaptive effect [14,15]. In spiny chromis *Acanthocromis polyacanthus* and sheepshead minnows *Cyprinodon variegatus*, thermal tolerance in the offspring was enhanced by parental exposure to warm temperatures [16,17].

Among the abiotic environmental variables affecting organisms, temperature is one of the most studied [18]; specifically in the last decade, with the changing climate manifesting in higher levels of short- and long-term fluctuations [19]. Across Europe for example, the Heat Wave Magnitude Index (HWMId, adimensional), expressing the duration and intensity of heatwaves, has increased steadily from an average of −2 to +2 between 1980 and 2015, and is projected to augment until 2100, even considering the most conservative of the predictions [20,21]. In riverine systems, global mean and maximum water temperatures are projected to increase by 0.8–1.6 and 1.0–2.2°C respectively according to the Special Report on Emissions Scenario (SRES) B1–A2 for 2071–2100 relative to 1971–2000 [22]. Considering these fluctuations there is a need to assess how and if organisms (and their gametes) can cope with such changes in their habitat, especially during sensitive life stages.

Organisms can respond in a multitude of ways to thermal variation, from changes in behaviour and phenology [23] to mitochondrial function [24,25] and osmoregulatory adaptations [26]. Importantly, temperature affects a key aspect defining the survival of a species—its ability to reproduce and attain fertilization success. Experimental evidence from a wide array of species highlights how temperature has damaging impacts on fertility [27] and that the threshold temperature for fertility is much lower than for survival [28]. In fact, high temperatures have been shown to affect the germ line and the associated reproductive efficiency of both sexes [29,30], because thermal stress affects meiotic divisions much more than mitotic proliferation [31,32].

Male and female gametes may respond differently in varying environments and their optimal conditions may vary [15], before and after ejaculation. Sperm production and function are particularly sensitive to higher temperatures which result in lower sperm density (e.g. [33], Cnidarian *Acropora digitifera*), lower motility (e.g. [34], *Drosophila subobscura*; [35], *D. tripunctata*), reduced testes size, sperm size and sperm competitiveness (e.g. [36], cowpea weevil *Callosobruchus maculatus*; [37], guppy *Poecilia reticulata*)*,* changes in biochemical composition (e.g. [38], several teleosts) and increased DNA damage (e.g. [29], *Mus musculus*; [39], *Sus* sp.). In the whitefish *Coregonus lavaretus*, the pre-fertilization incubation of sperm to warmer temperatures resulted in smaller offspring with reduced swimming performance as compared to the colder treatment [40].

Similarly, eggs are highly sensitive to temperature experienced before fertilization [41] where higher temperatures resulted in reduced production (e.g. [42], bed bug *Cimex lectularius*)*,* larger [15] (*T. castaneum*) or smaller egg sizes [43] (*C. maculatus*), reduced fertilization capability (e.g. [44], *Bos taurus*) and lower offspring fitness including lower survival rates (e.g. [14], *T. castaneum*; [45], fruit moth *Grapholita molesta*). External factors influencing gamete function could therefore provide the basis for the selection of adaptive responses in the resulting offspring. Such a process would allow individuals to improve their reproductive fitness by priming their gametes to the environment during fertilization and offspring development. Gamete thermal plasticity could evolve as an adaptive trait when the environment experienced by the parents matches that of the offspring [10]. However, the mechanisms by which temperature affects gametic performance and how these influence offspring fitness remain poorly understood [46–49].

Exploring the consequences of gametic thermal environments for offspring fitness is particularly important for stenothermal species like salmonids that are highly sensitive to thermal fluctuations [50–54]. Minor changes in the thermal environment experienced by sperm for instance have been found to have significant effects on its function in the common carp (*Cyprinus carpio*), in the brown trout (*Salmo trutta*) and in the greyling (*Thymallus thymallus*) and in the turbot *Lota lota* [55–58]. High temperatures can similarly cause increased production of reactive oxygen species (ROS) in human and mouse sperm that can lead to oxidative stress, disrupt the lipidic and proteic components of sperm and cause DNA damage [59,60].

We investigated the potential role of thermal plasticity and/or thermal selection in eggs or sperm and how this affects the resulting offspring and their adaptation to thermal variation. To do this, we monitored offspring survival to hatching, hatching synchrony, embryonic age at hatching and hatching success in crosses generated using warm- and cold-incubated gametes in Atlantic salmon (*Salmo salar*). These crosses were compared using a full-factorial design, with half of each clutch reared in cold temperatures and the other in warm temperatures. This specifically allowed us to test the hypothesis that the priming of gametes to warm or cold temperature before fertilization should benefit embryo development and hatching success in the same temperature and vice versa. In addition, we aimed to disentangle sex-specific effects using a full-factorial design and study offspring fitness in crosses where pre-fertilization temperatures of gametes were not matched.

## 2. Methods

### 2.1. Fish origin and gamete handling conditions

All Atlantic salmon used in this study came from the commercial strain Mowi from the breeding facility in Askøy (Norway). Stripping of gametes was conducted using standard hatchery procedures as described previously [61,62]. Briefly, gametes from males were collected from the urogenital pore by applying gentle abdominal pressure and prior to each stripping, urogenital pores were dried to avoid sperm activation before the start of the experiments. Eggs were instead collected from the abdominal cavity of euthanized females at the peak of their sexual maturation. Using these procedures, eggs from ten females and sperm from ten males were collected. Sperm samples were transferred into sterile flasks kept on ice in sealed polystyrene boxes. Eggs in their ovarian fluid were placed into sealed egg buckets and kept on ice. Gametes were then transported to Norwegian Institute of Marine Research (IMR) experimental aquaculture facilities and laboratories in Matre and processed the following morning.

### 2.2. Experimental design and gamete temperature priming

Following collection, gametes from ten males and females were split and incubated overnight for 24 h at either 2°C (cold) or 8°C (warm). The two temperatures used were selected because they are both within thermal the limits of Atlantic salmon [63]. *In vitro* fertilization assays were conducted following a full factorial design which resulted in four different treatments for each family (e.g. male 1 × female 1): warm sperm × warm eggs, warm sperm × cold eggs, cold sperm × warm eggs, cold sperm × cold eggs. In each fertilization assay 100 µl of raw milt were added to a batch of 200 eggs for each treatment and the mixture was subsequently activated with 200 ml of river water (5.7 ± 0.46°C). Each family was generated by a unique combination of male and female (e.g. male A × female A), and all individuals were only used for one cross (e.g. female A was only ever paired with male A, and vice versa). Each of the batches was split into two halves again after fertilization and one half reared in cold or warm hatchery rearing units kept at 2 ± 0.72°C or 8 ± 0.97°C respectively.

**Figure 1.**
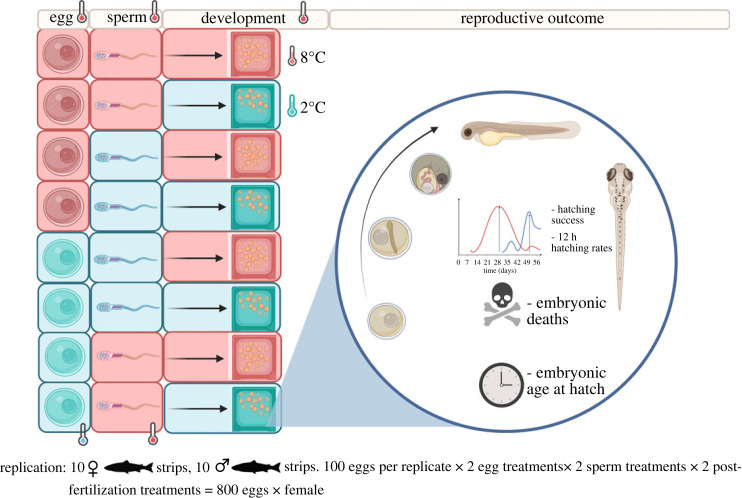
Experimental design showing the treatments (warm (red) or cold (azure)) for eggs, sperm and embryos in each unique family (*n* = 10). *In vitro* fertilization assays were performed by adding 100 µl raw milt to a batch of 200 eggs per treatment. Each of the batches was then split into two halves again after fertilization and reared in cold or warm hatchery conditions (100 fertilized eggs per family per treatment), using a full-factorial design. For each sub-clutch we assessed the reproductive outcome shown on the right including survival before and after the eyed stage, hatching success, hatching time and synchrony and developmental abnormalities.

For each sub-clutch, we assessed survival before and after the eyed stage, hatching success, hatching time and synchrony and developmental abnormalities (e.g. spinal deformities, incomplete neurogenesis, or abnormalities of the yolk sack; ‘malformed embryos’) every 12 h for the duration of the experiment (75 days for warm incubated and 180 days for cold incubated embryos; figure 1).

### 2.3. Statistical analyses

All analyses were performed using R Studio (RStudio (2021), Integrated Development for R; RStudio, PBC, Boston, MA) (v 1.3.1093) equipped with the packages reported in the electronic supplementary material. All data were analysed using linear mixed effect models (LMMs) and generalized linear mixed effect models (GLMMs) in the package *lme4*. Error distributions were determined by checking the relationship between variance and mean of the response variables as well as the necessary assumptions for data distribution [64]. Models were fitted using restricted maximum likelihood (REML) methodology for LMMs and maximum likelihood estimators for GLMMs, enabling model rectification and validation [65]. The residuals from linear models were also explored for normality and homoscedasticity. Significant main effects and interactions were extrapolated using *t*-tests with Satterthwaite's approximation controlling for degrees of freedom used as implemented in *lmerTest* and by using the functions available through *emmeans* and *emmtrends*. Additionally, the runs from the models were also generated in the form of analyses of variance with output from type III Wald Chi square tests. Model performance was explored for each dependent variable of interest by comparing residual dispersions, model predictions, AICs and BICs for each of the computed models through the *lme4* ‘summary’ function output and through residual diagnostics implemented by the package *DHARMa*. Fitting improvements between different hierarchical model structures were tested for significance by using the *anova* function.

### 2.4. Differences in embryonic age at hatching as a consequence of gamete priming and developmental temperature

Divergence in embryonic age at hatching among the thermal regimes was determined using *lmer* using a Gaussian family structure. Our fixed factors were egg and sperm pre-fertilization temperature and embryo development temperature (2 or 8°C) and all their possible interactions. Family ID was included as random factor with random slopes.

### 2.5. Hatching rates

Effects of temperature on hatching was analysed using the *glmer* function with binomial error structure (logit link) from the package *lme4*. This model included the proportion of offspring hatched relative to the starting number of eggs (*cbind*) entered as response variable, and time post-fertilization, egg and sperm pre-fertilization temperature and embryo development temperature and all their possible interactions as fixed effects. Family ID was included as random factor with random slopes.

### 2.6. Hatching success

Following the thermal treatments, differences in the proportion of successful hatchlings out of total egg numbers were modelled using *glmmTMB* with binomial error structure (logit link), for the better residual diagnostics that this model showed compared to other generalized linear models. We included the number of successfully hatched embryos on the total number of starting eggs (therefore accounting for the eggs that failed to develop) by using the *cbind* function and checked that the model was not over dispersed. Our fixed factors were egg and sperm pre-fertilization temperature and embryo development temperature and all their possible interactions. Family ID was included as random factor with randomized slopes.

### 2.7. Embryonic deaths and abnormal development

The number of dead embryos whose development was arrested after reaching the eyed stage (dead embryos at eyed stage), the number of embryos that died while hatching (dead embryos at hatching), and the number of hatched embryos with evident abnormalities were all analysed as proportions on the number of starting eggs for each treatment with the *cbind* function using *glmer,* function with binomial error structure (logit link) and optimized through the *‘Nelder-Mead’* function. Models included pre-fertilization temperatures for sperm and eggs respectively, embryo development temperature and their interactions as fixed effects and family ID (1 to 10) as random effect. To account for our full-factorial design, we added random slopes to account for variability across families.

## 3. Results

### 3.1. Effects on embryonic age at hatching

**Figure 2.**
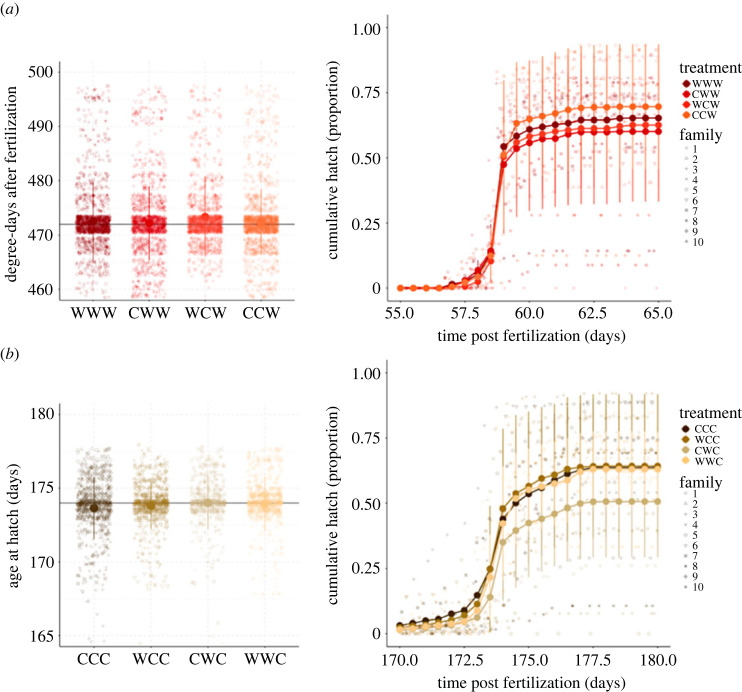
Age at hatching (left) and cumulative proportions of successfully hatched larvae (right) following the different combinations of gamete incubation regimes (cold (C), 2°C; warm (W), 8°C) in batches developing in the (*a*) warm (8°C) or (*b*) cold (2°C) embryo development treatment. Letters in the treatment acronyms are ordered as follows: egg temperature, sperm temperature, embryo development temperature. The means (dots) ± standard deviations (vertical bars) are shown for a total of 80 split-design crosses, with ten males and ten females crossed pairwise in an equal number of unique families.

Age at hatching was influenced by sperm pre-fertilization temperature. These effects where minor but significant and consistent across groups, with warm-incubated sperm leading to 0.74 degree-days (temperature (°C) × day) delay in the average emergence of hatchlings (table 1). However, there was a strong significant interaction between the sperm pre-fertilization temperature and embryo development temperature: when sperm pre-fertilization temperature matched embryo development temperature, larvae started hatching on average 1.02 degree-day earlier (figure 2*a*,*b*; table 1). On the contrary, matching eggs and developmental temperature led to an average 0.67 degree-days delay in hatching time. Embryo development temperature had the strongest effect with warm temperature resulting in development to be faster by 146 days compared to the cold incubated embryos (mean ± s.d. warm: 60.5 ± 0.709 days; cold: 173.7 ± 0.710).

**Table 1.** Linear mixed effect model (lmer in R) for age at hatching (degree days) in response to egg incubation temperature, sperm incubation temperature, and embryo development temperature. The results are shown for a total of 80 split-design crosses, with ten males and ten females crossed pairwise. Estimates are provided with standard error (SE), confidence intervals (CI) and degrees of freedom (df).

variable						
random					**variance**	
**Family ID**					1.47	
**Residual**					29.44	
Fixed	**Estimate**	**SE**	**CI**	**df1, df2**	***t***	***p***
**Intercept**	347.49	0.42	346.64, 348.36	1, 9.78	812.47	**<0.001**
**Egg**	0.326	0.27	−0.21, 0.86	1, 6134	1.17	0.23
**Sperm**	−0.74	0.29	0.16, 1.32,	1, 6133	2.52	**0.011**
**Dev**	125	0.27	−124.60, 125.66	1, 6131	460.99	**<0.001**
Egg*Sperm	−0.539	0.40	−1.33, 0.25	1, 6131	−1.33	0.182
Egg*Dev	0.672	0.38	−0.08, 1.42	1, 6133	1.74	**0.008**
Sperm*Dev	−1.024	0.40	−1.81, −0.23	1, 6132	−2.54	**0.011**
Egg*Sperm*Dev	−0.177	0.55	−1.27, 0.93	1, 6131	−0.31	0.749

### 3.2. Proportion of hatchlings born every 12 h

**Figure 3.**
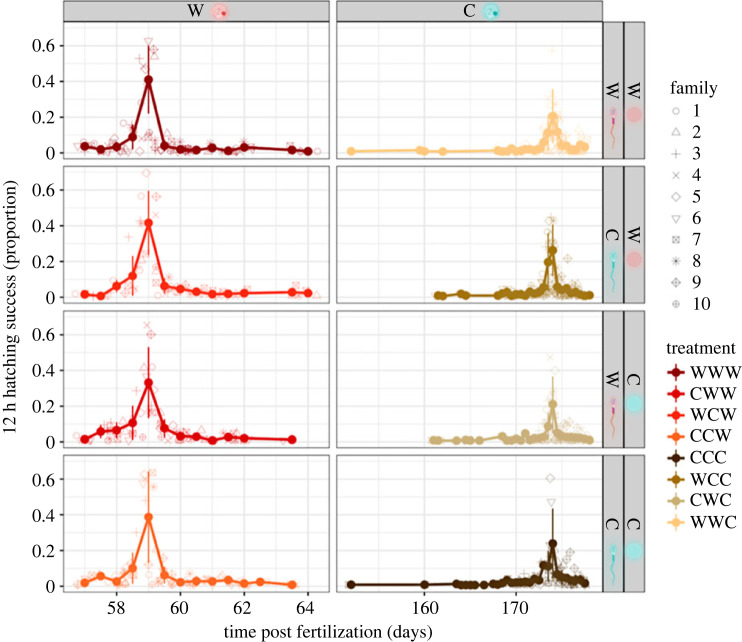
Hatching rates (proportion) assessed every 12 h under the different thermal regimes (cold (C), 2°C; warm (W), 8°C). Letters in the treatment acronyms are ordered as follows: egg temperature, sperm temperature, embryo development temperature. The means (dots) ± standard deviations (vertical bars) are shown for a total of 80 split-design crosses, with ten males and ten females crossed pairwise.

Hatching rate was influenced by gamete pre-fertilization temperature but showed opposite trends for egg and sperm. Hatching rates were on average 18 ± 5.1% higher in eggs exposed to warm pre-fertilization temperature but were significantly lower by 11 ± 5.5% when sperm were exposed to warm temperature. Three-way interactions and *post hoc* tests showed that when developmental temperature also matched sperm and egg temperature, hatching rate was 28 ± 11% lower. Developmental temperature showed again the greater impact with more than double the number of hatchlings every 12 h recorded in warm groups as compared to cold groups and explaining a variation through time of 66 ± 5.2% (figure 3, table 2).

**Table 2.** Generalized linear mixed effect model (glmer in R) for hatching rate in response to pre-fertilization temperature for eggs, sperm, and embryo development temperatures (2 or 8°C). The results are shown for a total of 80 split-design crosses, with ten males and ten females crossed pairwise and creating ten unique families (family ID = 10). Estimates are provided with standard error (SE), confidence intervals (CI), and degrees of freedom (df). Number of observations = 701.

variable						
random					**variance**	
**Family ID**					0.08	
**Number of obs:**	701	Groups:	FAMILY,	10		
Fixed	**Estimate**	**SE**	**CI**	**df1, df residuals**	***z***	***p***
**Intercept**	−2.96	0.09	−3.17, −2.76	1, 692	−30.28	**<0.001**
**Egg**	0.18	0.05	0.08, 0.29	1, 692	3.62	**<0.001**
**Sperm**	−0.11	0.06	−0.23, −0.01	1, 692	−2.13	**0.032**
**Dev**	0.67	0.05	0.56, 0.77	1, 692	−12.67	**<0.001**
Egg*Sperm	0.04	0.07	−0.10, 0.19	1, 692	0.55	0.58
Egg*Dev	0.07	0.07	−0.21, 0.07	1, 692	−0.91	0.36
Sperm*Dev	0.04	0.07	−0.11, 0.19	1, 692	0.55	0.57
Egg*Sperm*Dev	−0.28	0.10	−0.49, −0.08	1, 692	0.10	**0.007**

### 3.3. Hatching success

**Figure 4.**
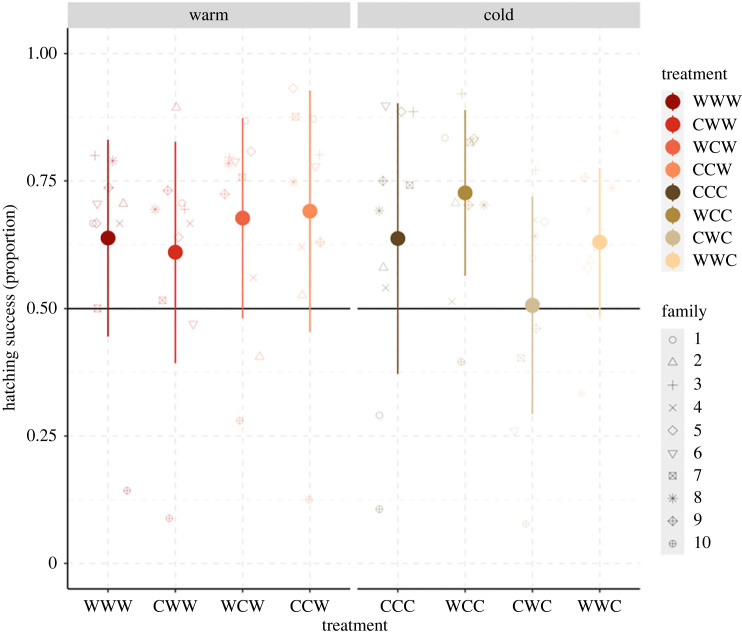
Proportion of successfully hatched embryos in response to the different thermal regimes at the pre- and post-fertilization stages (cold (C), 2°C; or warm (W), 8°C). Letters in the treatment acronyms are ordered as follows: egg temperature, sperm temperature, embryo development temperature. The data are shown as mean ± SE for a total of 80 split-design crosses, with ten males and ten females crossed pairwise.

Total hatching success showed opposite trends in response to pre-fertilization temperature for sperm and eggs, with warm-incubated sperm siring significantly lower numbers than warm-incubated eggs (a reduction in 20 ± 6.8% and an increase in 14 ± 6.6% respectively). We found no significant interaction between sperm and egg pre-fertilization temperature, but we found a negative effect when egg pre-fertilization temperature and embryo development temperature were set to 8°C (figure 4, table 3).

**Table 3.** Generalized linear model (glmer in R) for the proportion of successfully hatched embryos in response to pre-fertilization temperature for eggs, sperm, and embryo development temperatures (2 or 8°C). The results are shown for a total of 80 split-design crosses, with ten males and ten females crossed pairwise and creating ten unique families (family ID = 10). Estimates are provided with standard error (SE), confidence intervals (CI), and degrees of freedom (df). Number of observations = 78.

variable						
random					**variance**	
**Family ID**					0.08	
**Number of obs:**	78	Groups:	FAMILY,	10		
Fixed	**Estimate**	**SE**	**CI**	**df1, df residuals**	***z***	***p***
**Intercept**	−0.52	0.13	−0.79, −0.23	1, 9	−3.96	**<0.001**
**Egg**	0.14	0.06	0.01, 0.27	1, 70	2.11	**0.004**
**Sperm**	−0.20	0.06	−0.33, −0.07	1, 70	−2.94	**<0.001**
**Dev**	0.08	0.06	−0.04, 0.21	1, 70	1.29	0.15
Egg*Sperm	0.07	0.09	−0.12, 0.26	1, 70	0.74	0.32
Egg*Dev	−0.15	0.09	−0.34, 0.03	1, 70	−1.68	**0.009**
Sperm*Dev	0.08	0.09	−0.10, 0.27	1, 70	0.90	0.22
Egg*Sperm *Dev	−0.01	0.13	−0.27, 0.24	1, 70	0.07	0.94

### 3.4. Effect on embryonic deaths and developmental abnormalities

The proportion of embryos that died after reaching the eyed stage was significantly lower in the cold embryo development treatment than in the warm treatment, although in males gametes exposed to warm pre-fertilization temperature, the number of embryos dying after the eyed stage tended to be lower (non-significant). We found no effects of gamete pre-fertilization temperature on embryo survival after the eyed stage (electronic supplementary material, figure S1, table S2). Embryo development temperature, but not the pre-fertilization gamete temperature, affected the numbers of embryos dying during the hatching process as well as the embryos showing clear signs of abnormal development. Interestingly, these effects were detected exclusively in cold-developed groups (electronic supplementary material, figures S2 and S3; tables S3 and S4).

## 4. Discussion

Our study provides clear evidence that in an externally fertilizing species, the thermal environment experienced by gametes alone at the pre-fertilization stage can influence reproductive fitness. We further support the idea that thermal regimes affect gamete function [27,66–68] and found that the effect of the temperature experienced by gametes prior to fertilization was opposite in sperm and eggs. In addition, we found that temperature during embryo development was the main driver of differences in survival, age at hatching and developmental abnormalities, but it did not affect total number of offspring. The matching of gamete incubation temperature with embryo development temperature did not improve overall hatching success and again sperm and eggs showed opposite trends. We discuss our results and possible mechanisms that may explain the observed effects below.

### 4.1. Temperature effects on sperm and eggs

Sperm traits such as morphology, swimming behaviour, longevity and motility can be affected by environmental temperature [15,40,57]. In our study, sperm performance was negatively affected by warmer temperatures, translating into lower hatching success. These temperature effects on sperm can be explained by a series of non-mutually exclusive mechanisms. Sperm selection in response to varying environments could act in two ways. First, a specific gamete cohort could be selected that is particularly adapted to specific environmental conditions [69] and second, environmental conditions can hamper the biochemical and biomolecular pathways of gamete function with consequences for reproduction [49]. In our study, the warmer temperature of 8°C could increase ROS production in sperm [55] and their impact is known to reduce membrane fluidity, mitochondrial function, enzymatic activity and DNA integrity [60,70]. Similar changes have been shown in inactivated sperm in the closely related brown trout *Salmo trutta* [58]. Males in many taxa have been shown to vary sperm motility and phenotype in response to varying environmental conditions [71–73]. Such changes can be induced by a variety of abiotic environmental factors such as pH, ionic content and temperature [74]. Similar to sperm, egg quality is affected by environmental temperature and may cause variation in traits including size and biochemical composition of the yolk [15,75].

A particularly interesting finding was the opposite effect of temperature on sperm and eggs with eggs performing better at 8°C and sperm performing better at 2°C. This finding suggests that a global increase in temperature may be particularly important for male fertility, and the resulting offspring (e.g. in *Tribolium* flour beetles, see Sales *et al.* [14]). Such differences between males and females acquire relevance as salmon spawning events are characterized by a male-biased operational sex-ratio with more males competing for a smaller pool of females [76]. This reflects the intensity of breeding competition between males, as also shown by the evolution of alternative reproductive tactics [77,78] in this species [79,80] as well as in other salmonids [81]. Therefore, it is possible that warming-related effects could lower sperm competition dynamics and alter mate choice even more in an already threatened species. Post-release environmental temperature could also affect gamete function and phenotypic plasticity, as well as the interaction between male and female haplotypes in response to environmental fluctuations. However, we found no interaction effects between gamete pre-fertilization temperature and developmental environment that would support this idea (see next section).

### 4.2. Gamete effects on offspring fitness

Pre-fertilization temperature variation did not only affect gamete performance but also offspring fitness. Therefore, the pre-fertilization thermal environment could have shaped offspring fitness via epigenetic factors triggered by physiological changes [82,83], as well as via the selection of specific haplotypes as previously described [69]. Several epigenetic markers such as DNA methylation, modifications of histones or cytoplasmic and nuclear proteins could be responsible for the variation in embryonic age at hatching and hatching rates [84,85]. Interestingly, high ROS levels experienced by sperm have been linked to altered methylation patterns of the haploid DNA of sperm, as well as its lipidic and proteic content [59,60]. Furthermore, temperature can affect components of the seminal fluid including peptides, RNAs, enzymes and hormones which are known to influence offspring development [48,71,86–88]. Additionally, changes in the composition of reproductive fluids can in turn affect gamete composition and the resulting offspring fitness [89]. Such effects on sperm or seminal fluid components could have been altered in response to the thermal treatments performed in our experiment, leading to the observed lower hatching success observed among warm-exposed sperm groups. In the whitefish *Coregonus lavaretus*, offspring sired by sperm that had been exposed to 6.5°C pre-fertilization exhibited poorer swimming performance and were smaller than their siblings sired by sperm kept at 3.5°C [40]. Neither differences in sperm performance were observed between the two temperature regimes, nor were any effects on hatching success. One possible explanation for the differences between the *Coregonus* study and our own study could be the smaller temperature range used in the former, which may be less stressful for sperm than our treatments. In addition, these temperatures and their effects are likely to be highly species specific.

Survival and normal development of embryos were mainly affected by embryo development temperature whereas pre-fertilization gamete incubation temperature only showed an effect in interaction with the embryo development temperature. The number of dead embryos at the eyed stage was significantly higher in the warm-development temperature treatments and tended to be lower in groups where sperm had been exposed to warm pre-fertilization temperature. Surprisingly, malformed hatchlings and mortality during the hatching process were observed exclusively in cold-developed groups, suggesting a different purging mechanism toward sub-optimal phenotypes operated by cold developmental temperatures. Within these treatments, the number of abnormal embryos was generally higher when sperm were exposed to warm temperatures, although not statistically significant, thus providing some insight into the detrimental effects of warm pre-fertilization sperm incubation temperature. Environmental events can be carried over via sperm and negatively affect offspring fitness [14]. It would be interesting to investigate if such negative effects on sperm were at least partly responsible for the lower hatching success and whether they occurred before or during fertilization, or during early development stages rather than later in development. It is also possible that the strength of all these above-mentioned changes could have been stronger if we had used a larger number of mating pairs, thus augmenting the statistical power. The temperature used in our experiment during fertilization was intermediate and standardized across all treatments and we cannot exclude that the abrupt change in temperature at fertilization may differentially affect some sperm cohorts leading to intra-ejaculate selection.

In crosses where sperm pre-fertilization temperatures and embryo development temperature were set to warm (8°C), offspring hatched on average ∼1 degree day earlier. This could be consistent with the idea of a gamete-driven adaptive response or with a physiological optimization [90]. In salmon, timing of hatching and larval emergence are tightly correlated [91,92] and early emerging alevins can acquire competitive advantage due to prior residency, showing better growth and survival in the wild [93]. However, despite observing a statistically significant delay in hatching in the treatments where sperm did not match the embryo development temperature, these differences were so small they are unlikely to represent a competitive advantage. In a study testing the presence for additive genetic variation for egg development timing between wild and farmed salmon, differences in hatching times between 1 and 15 degree days were interpreted to be biologically irrelevant when compared to other factors such as the timing of spawning among females, which can vary by several weeks [94]. On the other hand, we also found higher numbers of hatchlings emerging every 12 h in groups where egg pre-fertilization temperature was set to warm, and the opposite effect was observed for male gametes. Thus, hatching was in general more synchronous when eggs were kept in a warmer environment. However, the beneficial effect of warm incubation of eggs was not enough to maintain a synchronized hatching peak when sperm were also exposed to warm temperatures and the embryos developed in a warm environment. This lack of synchrony could hamper offspring success, because spread-out timing of hatching may increase the *per capita* predation risk [95]. On the other hand, spreading hatching over time could be an efficient strategy to increase the chance to match a favourable environment. Therefore, further studies should focus on this aspect to better understand its biological relevance in nature.

From an applied angle, the application of a cold treatment to sperm and a warm treatment to eggs could prove a useful strategy to maximize offspring production. Thermal priming of gametes could be therefore used within the hatcheries, where synchronously maturing embryos could improve sustainability and be again a productive advantage.

Nevertheless, we cannot exclude that in relation to hatching times and synchrony, sharper environmental fluctuations at temperatures that are outside the optimal temperature range for salmon might change the observed patterns and be more realistic in a climate change context (e.g. sudden heatwave). This may be particularly important where rapid fluctuations in temperature occur, such as those characteristic of man-induced climate changes over the last two decades [96]. In thermally sensitive and cold-adapted species like salmonids, we might not expect adaptive mechanisms to be induced by high temperatures. This could affect their ability to balance the overall loss of reproductive outcome in warm environments, and therefore furnish an advantage to their offspring under environmental fluctuations. In the brook trout *Salvelinus fontinalis*, maternal and paternal temperatures modulate the methylation patterns in offspring which may drive their adaptation to predictable environments [97]. However, in this study, both females and males were exposed to cool and warm temperatures prior to fertilization, and their offspring was split and developed in both environments, development temperature having no effect on methylation. This could be in line with our finding of no effect of embryo development temperature on hatching success. It appears that the conditions during gametogenesis play a key role. Heat shock proteins are continuously expressed during spermatogenesis and are known to play a pivotal role in sperm development. It has in fact been proposed that adaptations to different thermal environments could be adaptively modulated during gonadal development so that sperm function can better withstand future challenges and be effective at reproduction in a warmer environment [98]. The fact that we detected such changes when exposing gametes alone, prior to their activation and after gametogenesis, suggests that such processes may not be strictly parentally transmitted adaptations, but that that gametes alone could be involved.

## 5. Conclusion

Our results highlight opposite effects of thermal regimes on eggs and sperm, where a warmer environment was beneficial for eggs but detrimental for sperm. These specific disadvantageous environments experienced by gametes hampered hatching success and the total number of offspring. The effects on the overall reproductive output were predominantly influenced by the conditions experienced pre-fertilization and priming gametes to future temperatures can benefit the offspring. Our findings revealed small changes in hatching time and synchrony, but not in overall reproductive output as a consequence of gamete priming to future embryonic environmental conditions, and suggest that populations might not be able to effectively buffer the effects of unpredictable temperature fluctuations on gametes. Moreover, we highlight that salmon males could be the most affected under such climatic fluctuations and that the detrimental effect of warm temperatures on male gametes alone is enough to reduce the overall reproductive outcome. From an applied angle, we suggest that the differential storage of male and female gametes prior to fertilization protocols could be used to ameliorate the reproductive outcome within the hatcheries by increasing their productive efficiency and thus their sustainability.

## Data Availability

All the material, the raw data files and the script have been uploaded on Dryad: https://doi.org/10.5061/dryad.wstqjq2s9 [99]. A version of the Rscript has been made available on the Dryad repository. Supplementary material is available online [100].
